# Identification of quantitative trait loci for yield traits and fine-mapping of *qGW4* using the chromosome segment substitution line-Z708 and dissected single-segment substitution lines

**DOI:** 10.3389/fpls.2025.1524770

**Published:** 2025-02-11

**Authors:** Kai Zhou, Jinjin Yu, Zhaopeng Yu, Chunyu Chi, Jialin Ren, Zhuowen Zhao, Han Zhang, Yinghua Ling, Changwei Zhang, Fangming Zhao

**Affiliations:** Integrative Science Center of Germplasm Creation in Western China Science City; Rice Research Institute, Academy of Agricultural Science, Southwest University, Chongqing, China

**Keywords:** chromosome segment substitution line, yield traits, QTL, qGW4, additive effect

## Abstract

Identifying quantitative trait loci (QTL) for yield traits using single-segment substitution lines (SSSL) is essential for both targeted breeding and functional analysis of key genes. Here, a wide-grain rice chromosome segment substitution line (CSSL), Z708, carrying four substitution segments from Jinhui35 in the genetic background of Xihui18, was used to identify the QTL associated with grain size. Seven QTL for yield-related traits (*qGW4*, *qRLW4*, *qGWT4*, *qGW5*, *qRLW5*, *qGWT5*, and *qGPP5*) were identified on the substitution segments of the fourth and fifth chromosomes of Z708. Subsequently, four SSSLs (S1-S4), which harbored 16 QTL for yield traits, were constructed using molecular marker-assisted selection. These lines (S1-S4) exhibited a significant increase in yield per plant compared to that of Xihui18. Among them, *qGW4*, which controls wide grains, belongs to a single dominant gene action in S1 based on the frequency distribution of grain width and chi-square test analysis. Finally, *qGW4* was fine-mapped to the interval of 80-kb (minimum) and 310-kb (maximum) using both traditional fine mapping and overlapping substitution mapping of the newly constructed secondary SSSLs (S5-S8). Within this interval, four previously unreported candidate genes were predicted.

## 1 Introduction

Rice is a staple food crop that provides nearly half the daily caloric intake for humans worldwide (Bin et al., 2023). Rice production plays an essential role in global food security (Rezvi et al., 2023). According to the Food and Agriculture Organization (FAO) statistics for 2023, China is the largest rice producer globally, accounting for approximately 28% of the total rice production. Increasing rice yields has become a key focus in meeting the demands of food security for the growing population (Shalmani et al., 2023). However, rice yield, as a complex trait, is composed of the number of grains per panicle, number of effective panicles per plant, and 1000-grain weight. The 1000-grain weight is determined by the grain size, including the length, width, length-to-width ratio, and filling rate of the grains. Thus, yield traits are typical quantitative traits controlled by multiple minor-effect genes (Kumari et al., 2023; Zhong et al., 2020; McKenzie et al., 1983). These complex traits have been divided into several discrete Mendelian factors through quantitative trait locus (QTL) mapping using numerous molecular markers (Ali et al., 2010; Maurer et al., 2017; Shabir et al., 2017).

Chromosomal segment substitution lines (CSSLs), each carrying one or a few specific marker-defined donor segments in the genetic background of the adapted cultivar (Surapaneni et al., 2017), can improve the accuracy of QTL mapping (Ando et al., 2008; Ookawa et al., 2016). A CSSL that carries a single substitution segment from a donor, is called a single-segment substitution line (SSSL). Many QTL detected by SSSLs can be directly used in breeding by design. Therefore, SSSL libraries serve as a valuable platform for breeding by design through target chromosome segment substitutions (Zhu et al., 2009; Li et al., 2019; Zhang et al., 2021a).

Currently, several genes related to rice yield traits have been cloned using CSSLs and other primary segregated populations. These genes are involved in various signaling pathways, including phytohormones, G-protein signaling, MAPK signaling, the ubiquitin–proteasome pathway, and transcriptional factors (Li et al., 2021). Genes for grain number include *Grain number 1a (Gn1a)*, *Grain Number per Panicle1 (GNP1)*, *Plant Architecture and Yield 1* (PAY1), Frizzy panicles(*FZP*), *Regulator of Grain Number1(RGN1)* etc. (Li et al., 2021; Huang et al., 2018; Li et al., 2022). *Gn1a* encodes cytokinin oxidase/dehydrogenase (OsCKX2), which degrades the phytohormone cytokinin to control rice grain number (Ashikari et al., 2005). *GNP1* encodes *GA20ox1*, which participates in GA biosynthesis (Zhao et al., 2015). *PAY1*, encoding a protein containing a peptidase S64 domain, which affects the transport activity of polar auxins and alters the endogenous distribution of indole acetic acid (IAA) (Wu et al., 2016). The COMPASS-like complex, formed by OsWDR5a and OsTrx1, promotes flowering and panicle branching by modulating H3K4me3 levels, further highlighting its critical role in rice yield regulation (Jiang et al., 2018). *FZP* regulates rice secondary branches and grain numbers, a 4-bp tandem repeat deletion about 2.7 kb upstream of *FZP* affect the binding activities of auxin response factors to the *FZP* promoter, decrease *FZP* expression and increase secondary branches and grain yield in cultivated rice. In addition, OsPTB1/2 can mediate *FZP* translational repression by interacting with CUREs in the 3′ UTR of *FZP* mRNA, leading to changes in the NSB and GNP (Huang et al., 2018; Chen et al., 2022). *RGN1* regulates lateral grain formation by controlling *LOG*, a key gene in cytokinin biosynthesis. The favorable allele RGN1^C^ from wild rice promotes longer panicles and higher grain yield (Li et al., 2022). *DEP1* is a gain-of-function mutation causing truncation of a phosphatidylethanolamine-binding protein-like domain protein. In addition, the G- protein βγ subunits of *DEP1* also regulates grain size by interaction with MADS-domain transcription factors in rice. Furthermore, *DEP1-GNA* is a regulating module for rice panicle development, which is important to enhance rice yield (Huang et al., 2009; Liu et al., 2018; Zhang et al., 2025). Genes associated with grain size include *GS3, SMG1, GW2, GW5/qSW5, TGW6, qRBG1*, and *GLW7* etc. *GS3* encodes the G-protein γ subunit and negatively regulates grain length (Fan et al., 2006). *SMG1/MITOGEN-ACTIVATED PROTEIN KINASE KINASE4 (OsMKK4)* controls rice grain size by participating in the mitogen-activated protein kinase (MAPK) signaling pathway (Duan et al., 2014). *GW2* and *GW5/qSW5* control rice grain size by participating in the ubiquitination pathway (Song et al., 2007; Shomura et al., 2008; Weng et al., 2008). Furthermore, the GW2-WG1-OsbZIP47 pathway coordinates grain growth through ubiquitination and transcriptional repression, highlighting its role in controlling cell proliferation (Hao et al.,2021). *GW5* regulates grain width also via the brassinosteroid (BR) signaling pathway (Liu et al.,2017). *TGW6*, which encodes an IAA-glucose hydrolase, regulates grain size via the phytohormone pathway and negatively regulates grain weight (Ishimaru et al., 2013). *TGW2* encodes CELL NUMBER REGULATOR 1 and regulates rice grain width and weight by influencing cell proliferation and expansion in glumes (Ruan et al., 2020). *TGW3* regulates rice grain size by phosphorylation of OsIAA10-OsARF4 mediated auxin signaling (Ma et al., 2023). *OsNLP3 *forms the OsNLP3-OsCEP6.1 and OsNLP3-OsNF-YA8 modules, enhance grain weight (Sun et al., 2024). Natural variation in the promoter of *qRBG1/OsBZR5* enhances rice yield via the BR pathway (Zhang et al., 2024). *GLW7* encodes the transcription factor *OsSPL13* in rice, whose higher expression increases grain length and weight by promoting cell elongation (Si et al., 2016). The genes associated with tiller number include *Monoculm1 (MOC1)* and other related genes. *MOC1* encodes a plant-specific GRAS-family nuclear protein that acts as a vital regulator for controlling the formation of tiller buds (Li et al., 2003).

Although many genes for yield trait have been identified, our understanding of the mechanisms governing these traits remains incomplete. Numerous minor-effect QTL for yield traits still need to be identified. Therefore, identifying these minor QTL using SSSLs is crucial. In this study, we present the fine mapping of *qGW4* and the identification of QTL for yield traits using CSSL-708 and the developed SSSLs. We further demonstrate that Z708 carries a 4-segment substitution from Jinhui35 in the Xihui18 background. These findings are essential for exploring previously unknown genes for yield traits and providing ideal breeding germplasms for breeding by design.

## 2 Materials and methods

### 2.1 Plant materials

**Figure 1:**
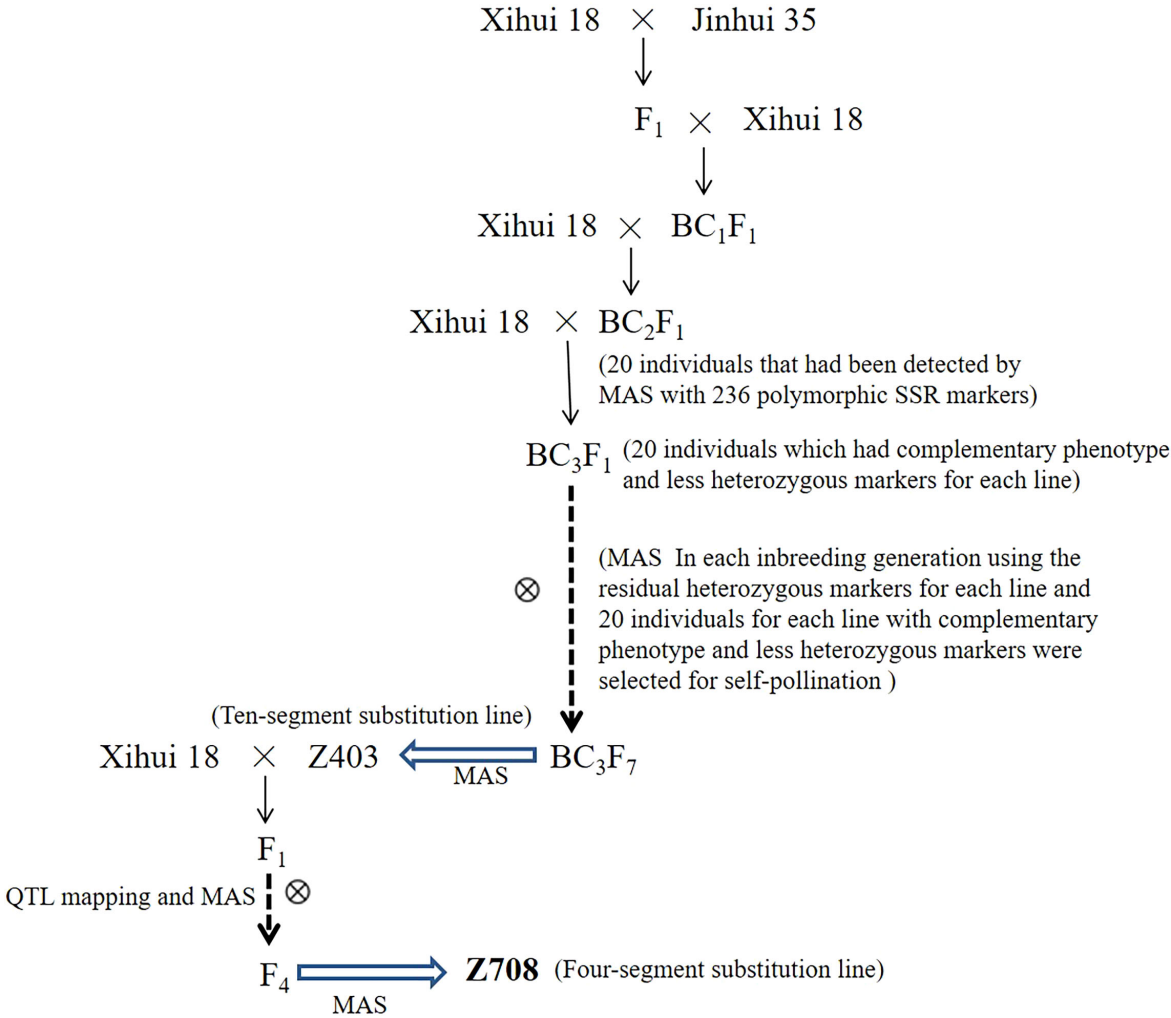
Flow chart of Z708 development.

The wide-grained CSSL-Z708 was used as the primary material. Z708 was developed by crossing the progeny (F_4_) of the recipient parents Xihui18 and Z403 using marker-assisted selection (MAS). Z403 was found to contain 10 substitution fragments from the donor Jinhui35 in the Xihui18 background, which was developed from Xihui18 as the recipient parent and Jinhui35 as the donor parent by advanced backcrosses in combination with single-sequence repeat (SSR) MAS from the BC_2_F_1_ to BC_3_F_7_ generations. Specific construction methods used for the development of Z403 have been described previously (Xu et al., 2023). Xihui18 and Jinhui35 are *indica* rice restorer lines bred by Southwest University, China. The Z708 chromosome substitution segment was identified as described previously (Sun et al., 2022). The specific flow chart of the genetic material construction was showed in the flow chart (**Figure 1**). The estimated length of the substitution segment was calculated as described previously (Paterson et al., 1991).

Because differences were found in only four chromosomal segments between its recipients Xihui18 and Z708, the material of the QTL mapping population was a secondary F_2_ population of 150 individuals constructed using Xihui18/Z708.

### 2.2 Planting methods

In July 2020, Xihui18 was crossed with Z708 and the hybrid seeds were harvested at the experimental station (Xiema town, Beibei distinct at 106.38° east longitude and 29.76° north latitude) of Southwest University, Chongqing, China. In September of the same year, the hybrids were sown at the Lingshui base (109.86° east longitude and 18.42° north latitude) in Hainan Province, China, and the F_1_ seeds were harvested. In March 2021, the parent seeds of Xihui18, Z708, Jinhui35, and the F_2_ population were sown in a field at the Rice Research Institute of the Southwestern University of Chongqing, China. In April 2021, 30 seedlings of Xihui18, Jinhui35, and Z708 and 150 individuals from the F_2_ population were transplanted to the same field, with 26.4 cm spacing between rows, 16.5 cm between hills, and 10 plants per row. In March and April 2022, 30 seedlings each of Xihui18 and Z708 and four F_2_ individuals for the development of SSSLs were cultivated and transplanted in the same field in Chongqing. Further, an F_3_ population was sown for the fine-mapping of *qGW4*, and all plants were transplanted into the same field in Chongqing. In March and April 2023, four SSSLs for QTL identification, as well as Xihui18 and Z708 were sown and transplanted (30 individuals for each line) in Chongqing. Additionally, four secondary heterozygous F_3_ individuals for the development of SSSLs and one NCL (all lanes of markers were the same as Xihui18) were cultivated for overlapping substitution mapping of *qGW4* (100 plants for each line). Field management was the same as local standard practices.

### 2.3 Measurement of agronomic traits

At the maturity stage, 10 plants each of Xihui18 and Z708 and 150 F_2_ plants were harvested. The following eight yield traits for yield-related traits were measured: plant height (PH), panicle number per plant (PN), panicle length (PL), grains per panicle (GPP), 10-grain length (GL), 10-grain width (GW), 1000-grain weight (GWT), and yield per plant (YD). The ratio of length to width (RLW) was calculated by dividing the grain length by grain width as described by Hui (Hui et al., 2020). Finally, simple statistical analyses, including the determining the mean value of each trait and the standard deviation, Student’s *t*-test for comparison between eight traits between Xihui18 and Z708, frequency distribution analysis of grain width in the F_3_ population, and the chi-square test, were performed using the statistical functions in Microsoft Excel 2016.

### 2.4 QTL mapping

The DNA of the parental plants and 150 F_2_ individuals used for QTL mapping was extracted using the CTAB method described previously (McCouch et al., 1988). PCR amplification, non-denaturing polyacrylamide gel electrophoresis, and rapid silver staining were performed, as described previously (Zhao et al., 2016). The Xihui18-type band was scored as ‘-1,’ the Z708-type band was scored as ‘1,’ the heterozygote was scored as ‘0,’ and a missing band was scored as’.’. The specific description of QTL mapping using the mixed linear model (MLM) method in SAS 9.3 software was the same as that described in a previous study (Zhao et al., 2016). The threshold for determining whether a QTL existed was set at p < 0.05.

### 2.5 Development of SSSLs

Based on the QTL mapping information, four F_2_ individuals carrying only one single target substitution segment and 0-1 heterozygous markers for each line were selected and planted as Z1229, Z1231, Z1232, and Z1234 in 2023, with 30 plants per line. Then, the leaves of 20 individuals were taken from each line to construct SSSLs using MAS with the target substitution markers and residual heterozygous markers. Finally, the homozygous SSSLs (S1, S2, S3, and S4) were screened.

### 2.6 Identification and analysis of the additive effect of QTL for yield traits using four SSSLs

At maturity, 10 plants each of Xihui18 and SSSLs (S1-S4) were harvested. Eight yield traits were measured: grain length, grain width, ratio of length to width, 1000-grain weight, panicle number per plant, panicle length, grain number per panicle, and yield per plant. The specific method has been described by Xu and Sun (Xu et al. 2023; Sun et al. 2022). For each SSSL (S1-S4), QTL were identified using one-way analysis of variance (ANOVA) and least significant difference (LSD) multiple comparisons with Xihui18 for each SSSL_i_ in IBM SPSS Statistics 25.0. At *p*-value < 0.05, a QTL for a certain trait was considered to exist in the SSSL_i_. The genetic model in a certain environment was *p_0_* = *μ_0_* + *ϵ* for Xihui18 and *p_i_* = *μ_0_* + *a_i_* + *ϵ* for an SSSL carrying a specific QTL (*p_0_* and *p_i_* represented the phenotypic value of any plant in a plot of xihui18 and the SSSL_i_, *μ_0_* represented the mean value of the Xihui18 population, *a_i_* represented the additive effect of the QTL). Thus, *a_i_* was equal to half of the difference between *p_i_* and *p_0_* (half was estimated as the genetic effect).

### 2.7 Inheritance analysis, fine-mapping, and overlapping substitution mapping of *qGW4*

An F_3_ population comprising 285 individuals developed from an F_2_ recombinant plant of *qGW4* was used for *qGW4* inheritance analysis and fine mapping. Among them, 74 recessive individuals (narrow grains) and seven newly designed polymorphism SSR markers together with RM3276, were utilized to analyze the linkage with *qGW4*. Moreover, four individuals with different genotypes were screened to construct secondary SSSLs of *qGW4* in the F_3_ generation. Furthermore, the grain widths of all SSSL individuals and one NCL population (10 individuals) (all lanes of markers were the same as Xihui18) were measured to be utilized for the overlapping substitution mapping of *qGW4*. The specific method has been described previously (Yang et al., 2021). When the grain width differed significantly between a secondary SSSL and Xihui18, the QTL controlling grain width was located in the substitution segment of the SSSL. When multiple substitution segments in the SSSLs overlapped with the grain width, the QTL was mapped to the overlapping region.

## 3 Results

### 3.1 Detection of substitution segments in CSSL-Z708

**Figure 2:**
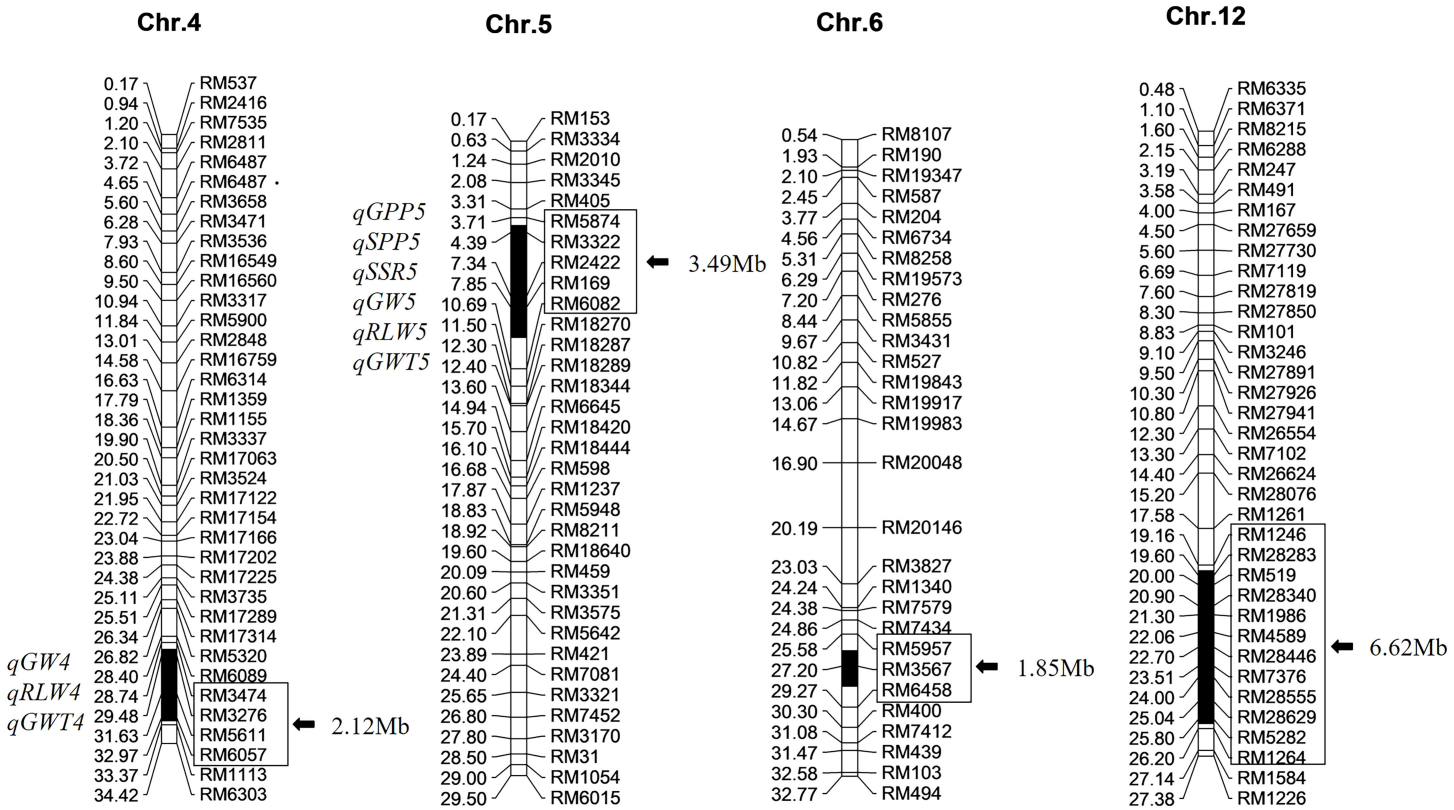
Chromosome substitution segments and the harbored QTL of Z708. The reference genome is indica rice cultivar ‘9311’. The physical distances (Mb) and QTL are marked on the left of each chromosome, the markers and lengths of the substitution segments exhibit on the right. Black sections on each chromosome indicate the substitution segments.

Based on the previous breeding of Z708 with 236 polymorphic SSR markers between two parents selected from 429 markers covering the whole rice genome 16 SSR markers in four substitution fragments and 36 outside them from 12 chromosomes were used to detect the accuracy of the substitution segments and the purity of genetic background in ten plants of Z708. The substitution segments of the 10 individuals of Z708 were identical, and no other residual fragments from Jinhui35 were detected. These results confirm the accuracy of the genotype in CSSL-Z708. Z708 contained four substitution segments from Jinhui35 in the Xihui18 background with a total length of 14.08 Mb and an average substitution length of 3.52 Mb, which were distributed on chromosomes 4, 5, 6, and 12 (**Figure 2**).

### 3.2 Phenotype analysis of Z708 and Xihui18

**Figure 3:**
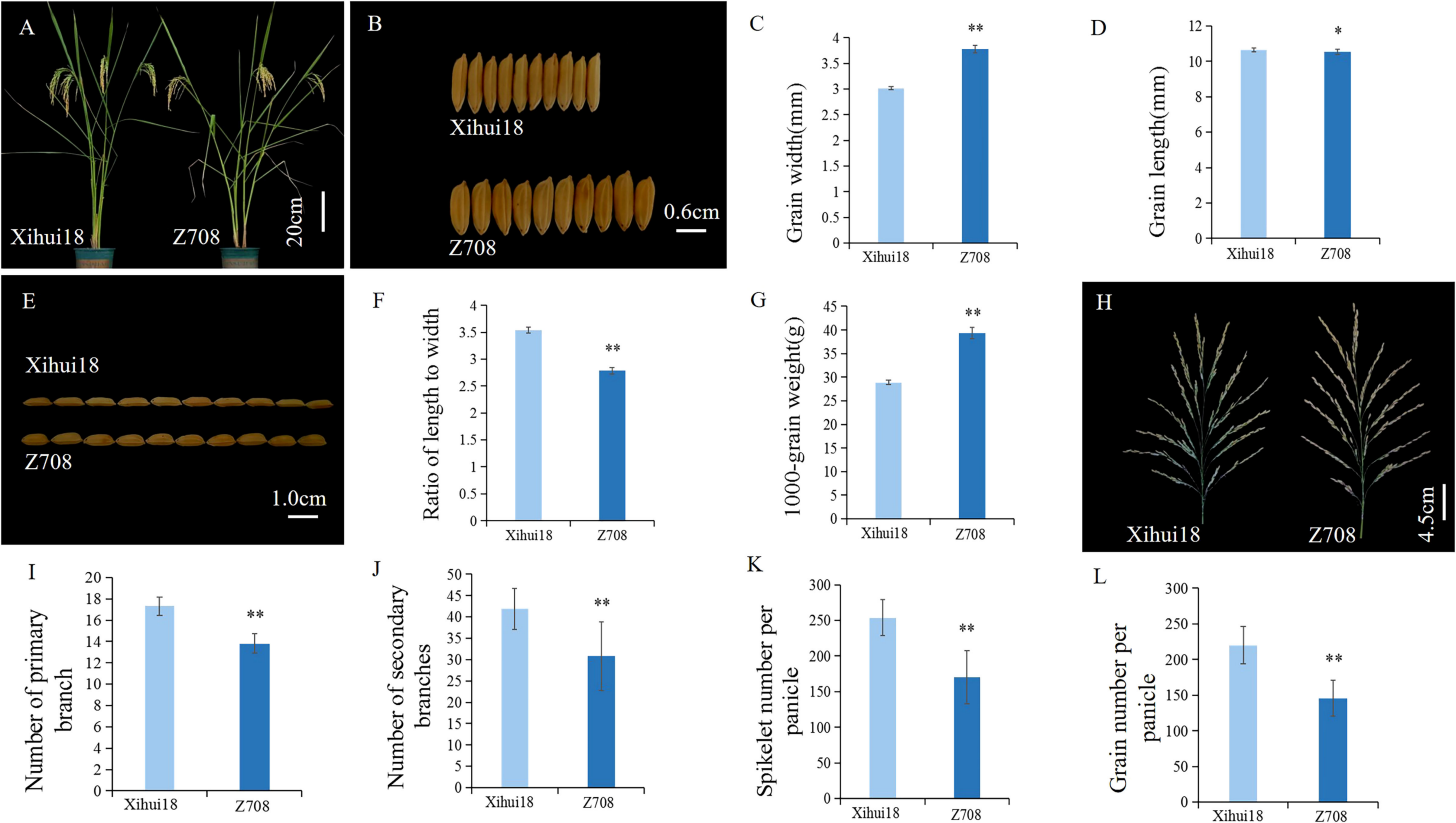
Plant-type and grain size analysis of Xihui18 and Z708. **(A)** The phenotype of Xihui18 and Z708 at the milky stage. **(B, C)** Grain width of Xihui18 and Z708. **(D, E)** Grain length of Xihui18 and Z708. **(F)** Ratio of length to width, **(G)** 1000-grain weight. **(H)** spike shape of Xihui18 and Z708. **(I)** Number of primary branch. **(J)** Number of Secondary branches. **(K)** Spikelet number per panicle. **(L)** Grain number per panicle. * and ** represent differences at the 0.05 and 0.01 levels, respectively.

As only four substitution segments differed from Xihui18, Z708 was considered a near-isogenic line (NIL) relative to Xihui18. The Z708 plant type (**Figure 3A**) resembled that of Xihui18, but still exhibited a heavy-spike phenotype. The most attractive characteristics of Z708 were its large grain size (**Figures 3B–G**) and less grain number (**Figures 3H–L**, **Table 1**). Compared to Xihui18, the grain width of Z708 increased significantly by 25.48% to 3.78 mm (**Figures 3B, C**, **Table 1**), while the 1000-grain weight (39.38 g) of Z708 increased by 36.31% (**Figure 3G**). In contrast, the grain length (10.54 mm) (**Figures 3D, E**) and the ratio of length to width (**Figure 3F**, **Table 1**) in Z708 were significantly lower, by 1.12% and 21.20%, respectively. Additionally, the number of primary branches (13.81), secondary branches (30.82), spikelets per panicle (170.18), and grains per panicle (145.75) of Z708 decreased significantly by 20.27%, 26.50%, 32.96%, and 33.79%, respectively, compared to those in Xihui18 (**Figures 3I–L**, **Table 1**).

### 3.3 QTL for yield traits harbored in the substitution segments of Z708

Using an F_2_ segregated population (the specific parameters in **Table 1**) from Xihui18/Z708 for QTL mapping, nine QTL were detected on chromosomes 4 and 5, including six for grain size and three for grain number related traits (**Table 2**). The wide grain of Z708 was controlled by *qGW4* and *qGW5*, both with additive effects of 0.08 mm, which explained 17.17% and 15.32% of the phenotypic variation, respectively. The additive effects of *qRLW4* and *qRLW5* from Jinhui35 reduced ratio of length to width of Z708 by 0.09 and 0.07, respectively, explaining the variation of 24.94% and 16.37%, respectively. The additive effects of *qGWT4* and *qGWT5* from Jinhui35 alleles enhanced the 1000-grain weight of Z708 by 1.13 g and 1.15 g, explaining the variation of 16.23% and 16.86%, respectively. The less grain numbers of Z708 was resulted from *qGPP5, qSPP5* and *qSSR5* from Jinhui35, whose additive effect reduced 9.54 grains per panicle, 8.30 spikeletes per panicle and 1.25 pencent points of seed setting rate in Z708, explaining 4.04%, 2.68% and 2.11% of the according variation, respectively (**Table 2**). 

**Table 1:** Yield traits of Xihui18, Z708 and the F_2_ population.

Traits	Mean ± SD (Parents)		F_2_ population			
	Xihui18	Z708	Mean±SD	Range	Skew	Kurt
Number of primary branches	17.32±0.85	13.81±0.90**	15.20±1.14	11.17-17.71	-0.39	0.55
Number of secondary branches	41.93±4.80	30.82±8.03**	41.04±8.84	18.00-64.57	-0.02	-0.13
Spikelets per panicle	253.86±25.10	170.18±37.12**	221.29±51.388	40.67-365.00	-0.22	0.99
Grains per panicle	220.16±26.00	145.75±25.36**	193.52±48.2	17.33-328.25	-0.48	1.60
Seed setting rate (%)	86.87±3.72	86.19±4.86^NS^	86.92±8.63	42.21-95.42	-3.03	11.1
Grain length (mm)	10.66±0.10	10.54±0.13*	10.27±0.19	9.80-10.70	0.02	-0.40
Grain width (mm)	3.01±0.03	3.78±0.07**	3.18±0.22	2.80-4.00	0.99	0.79
Ratio of length-width	3.54±0.06	2.79±0.06**	3.24±0.21	2.61-3.60	-0.77	-0.08
1000-grain weight (g)	28.89±0.50	39.38±1.20*	31.42±3.17	25.10-40.70	0.83	0.28

* and ** indicate a significant difference between the two parents at *P* < 0.05 and *P* < 0.01, respectively.

**Table 2:** QTL for yield-related traits carried by the substitution fragments of Z708.

Trait	QTL	Chr.	Marker interval	Additive effect	Var. (%)	*P*-value
Grain width (mm)	*qGW4*	4	RM3276-RM5611	0.08	17.17	0.0009
	*qGW5*	5	RM3322-RM169	0.08	15.72	<0.0001
Ratio of length to width	*qRLW4*	4	RM3276-RM5611	-0.09	24.94	<0.0001
	*qRLW5*	5	RM3322-RM169	-0.07	16.37	<0.0001
1000-grain weight (g)	*qGWT4*	4	RM3276-RM5611	1.13	16.23	0.0013
	*qGWT5*	5	RM3322-RM169	1.15	16.86	<0.0001
Grains per panicle	*qGPP5*	5	RM3322-RM169	−9.54	4.04	0.0079
Spikeletes per panicle	*qSPP5*	5	RM3322-RM169	-8.30	2.68	0.0238
Seed setting rate (%)	*qSSR5*	5	RM3322-RM169	-1.25	2.11	0.0376

### 3.4 Construction of SSSLs and identification of QTL for yield traits

**Figure 4:**
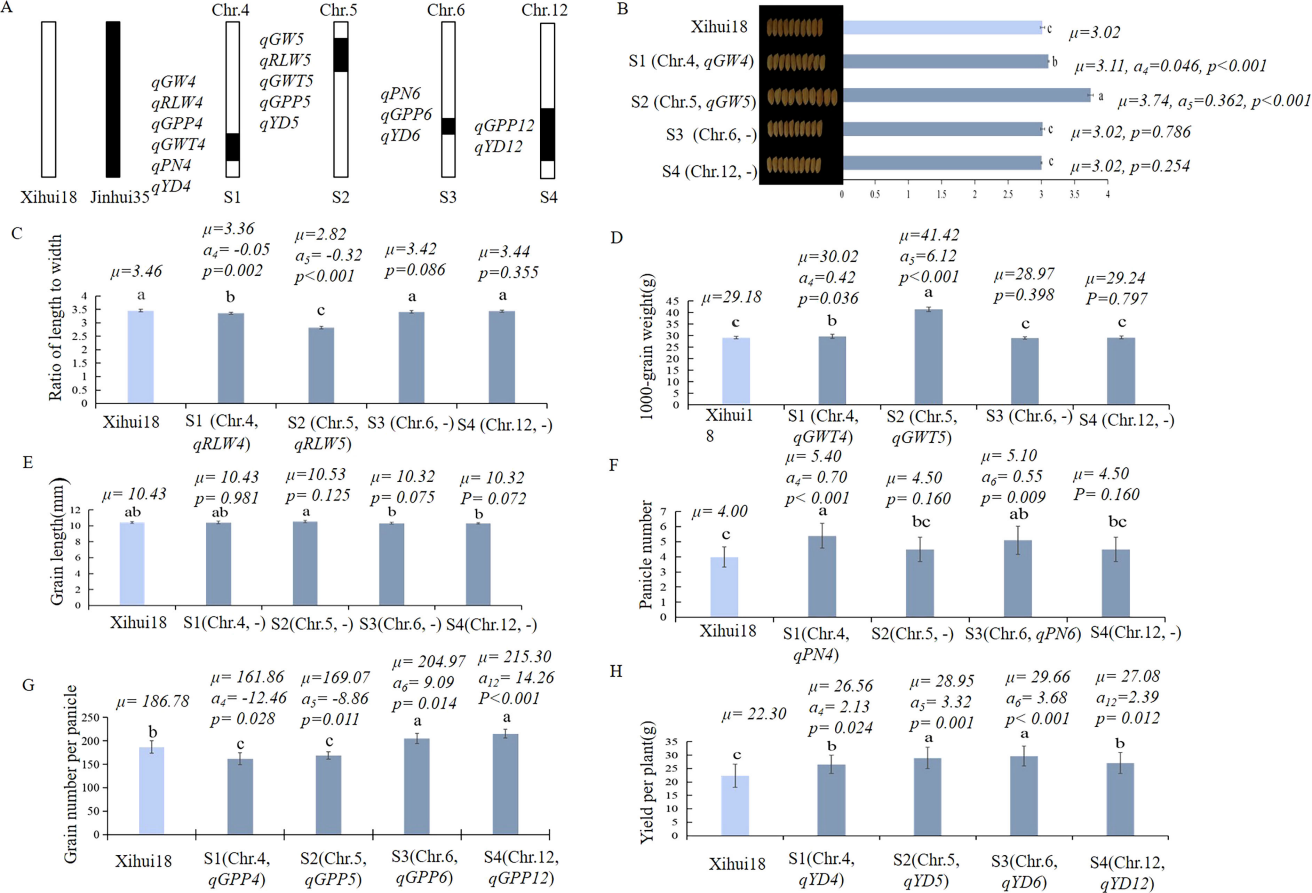
Construction of SSSL dissected from Z708 and analysis of additive effects of QTL for yield traits within them. **(A)** Diagram of the substitution segments and QTL in S1-S4. B-L: The QTL parameters in the different SSSLs. **(B)** Grain width; **(C)** Ratio of length to width; **(D)** 1000-grain weight; **(E)** Grain length; **(F)**: Panicle number per plant; **(G)** Grain number per panicle; **(H)** Yield per plant. Different lowercase letters represent significant difference (p < 0.05), which determined by Duncan’s multiple comparison. *μ*: the average value of each line; *a*: additive effect for each QTL controlling the trait. S1 (Chr.4: RM3476 (28.74 Mb)–RM3276 (29.48 Mb)-RM5611 (31.63 Mb)–RM6057 (32.97 Mb)); S2 (Chr.5: RM5874 (3.71 Mb)–RM3322 (4.39 Mb)-RM2422 (7.34 Mb)-RM169 (7.85 Mb)–RM6082 (10.69 Mb)); S3 (Chr.6: RM5957 (25.58 Mb)–RM3567 (27.20 Mb–RM6458 (29.27 Mb)); S4 (Chr.12: RM1246 (19.16 Mb)– RM1986(21.30Mb)-RM2855 (24.0Mb)-RM5282 (25.80Mb)–RM1264 (26.20 Mb)). The middle markers connected with hyphens represent the substitution fragment from the donor, while the markers of the border linked with”–”indicate that recombination might have occurred.

Four SSSLs (S1, S2, S3, and S4) were developed using marker-assisted selection (MAS) based on the QTL mapping results (**Figure 4A**). The single-segment substitution line S1 harbored the substitution segment RM3476–RM3276-RM5611–RM6057 of chromosome 4. S2 carried the substitution segment RM5874–RM3322-RM2422-RM169–RM6082 of chromosome 5. S3 contained the substitution segment RM5957–RM3567–RM6458 of chromosome 6. S4 harbored the substitution segment RM1246–RM1986-RM2855-RM5282–RM1264 of chromosome12. The seven QTL identified above were validated using S1 and S2. Further, nine QTL for yield traits were detected by S1, S2, S3, and S4, including *qGPP4*, *qGPP6* and *qGPP12* for grain number per panicle; *qYD4*, *qYD5*, *qYD6* and *qYD12* for yield per plant; and *qPN4* and *qPN6* for panicle number per plant (**Figure 4A**).

The grain width (3.11and 3.74 mm) of S1 carrying *qGW4* (a = 0.05 mm) and S2 containing *qGW5* (a =0.36 mm) was significantly wider than that of Xihui18 (3.01 mm), whereas S3 and S4 without QTL for *GW* showed no significant differences in grain width (3.02 and 3.02 mm) from Xihui18 (3.01 mm) (**Figure 4B**). The ratios of the length to width (3.36 and 2.82) of S1 harboring *qRLW4* (a = −0.05) and S2 with *qRLW5* (a = −0.32) were significantly less than those (3.46, 3.42 and 3.44) of the recipient parent Xihui18, S3, and S4 without QTL for RLW (**Figure 4C**). The 1000-grain weights (30.02 and 41.42 g) of S1 carrying *qGWT4* (a = 0.42 g) and S2 with *qGWT5* (a = 6.12 g) were significantly higher than those of Xihui18, S3, and S4 (29.18, 28.97, and 29.24 g, respectively) without QTL for GWT (**Figure 4D**). The grain length of S1-S4 without QTL for this trait was the same as that of Xihui18 (**Figure 4E**).

The panicle number per plant (5.4 and 5.1) in S1 *qGW4* harboring *qPN4* (a = 0.70) and S3 carrying *qPN6* (a = 0.55) was significantly higher than that of Xihui18 (4.00) and those of S2 and S4 (4.50 and 4.50, respectively) without QTL for PN (**Figure 4F**). Grain numbers per panicle (204.97 and 215.30) of S3 containing *qGPP6* (a = 9.09) and S4 carrying *qGPP12* (a = 14.26) were significantly greater whereas those of S1 containing *qGPP4* (a = -12.46) and S2 with *qGPP5* (a = -8.86) (161.86 and 169.07, respectively) were significantly less than that (186.79) of Xihui18 (**Figure 4G**). The yield per plant of S1 with *qYD4* (a = 2.13 g), S2 carrying *qYD5* (a = 3.27 g), S3 containing *qYD6* (a= 3.68 g), and S4 harboring *qYD12* (a= 2.39 g) (26.56, 28.85, 29.66, and 27.08 g, respectively) was significantly higher than that of Xihui18 (22.30 g) (**Figure 4H**).

### 3.5 Genetic analysis of *qGW4* for grain width

**Figure 5:**
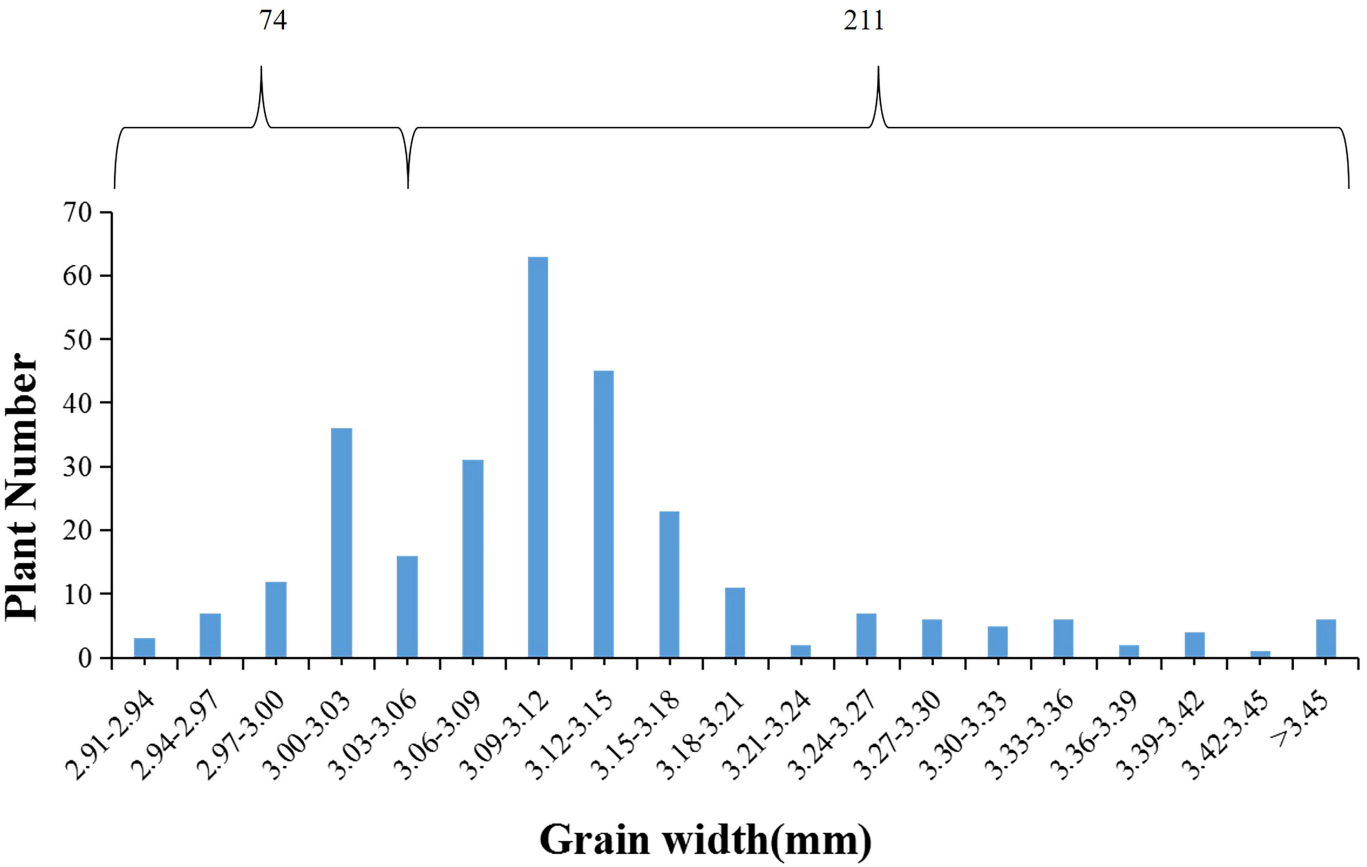
Frequency distribution of grain width in F_3_ population (285 plants) derived from a recombinant individual of *qGW4*.

The donor parent Jinhui35 exhibited a wide grain type, whereas the recipient parent Xihui18 displayed a narrow grain type. Z708, which carried four substitution fragments from Jinhui35 in a Xihui18 background, exhibited broad grains. In the F_3_ population consisting of 285 individuals constructed using a *qGW4* recombinant plant, the frequency of grain width displayed a bimodal distribution, one peak for a narrow grain type (from 2.91 mm to 3.06 mm), with 74 plants, and the other for broad grain type (from 3.06 to 3.45 mm), with 211 individuals (**Figure 5**). The chi-square test indicated that the broad-grain plants (211) and thin-grain individuals (74) fitted a separation ratio of 3:1 (χ^2^ = 0.17 < χ^2^_(0.05, 1)_ = 3.84) (**Table 3**). These results suggested that *qGW4* controlling wide grains from Jinhui35 in S1 displayed a single dominant gene action (**Figure 5**).

**Table 3:** The chi-square test of *qGW4* in the F_3_ population.

Trait	O(observed numbers)	E(theoretical number)	(|O-E|-1/2)^2^	(|O-E|-1/2)^2^/E	χ^2^
Wide grain	211	213.75	5.06	0.02	0.09
Narrow grain	74	71.25	5.06	0.07	

### 3.6 Fine-mapping of *qGW4* controlling wide grains

**Figure 6:**
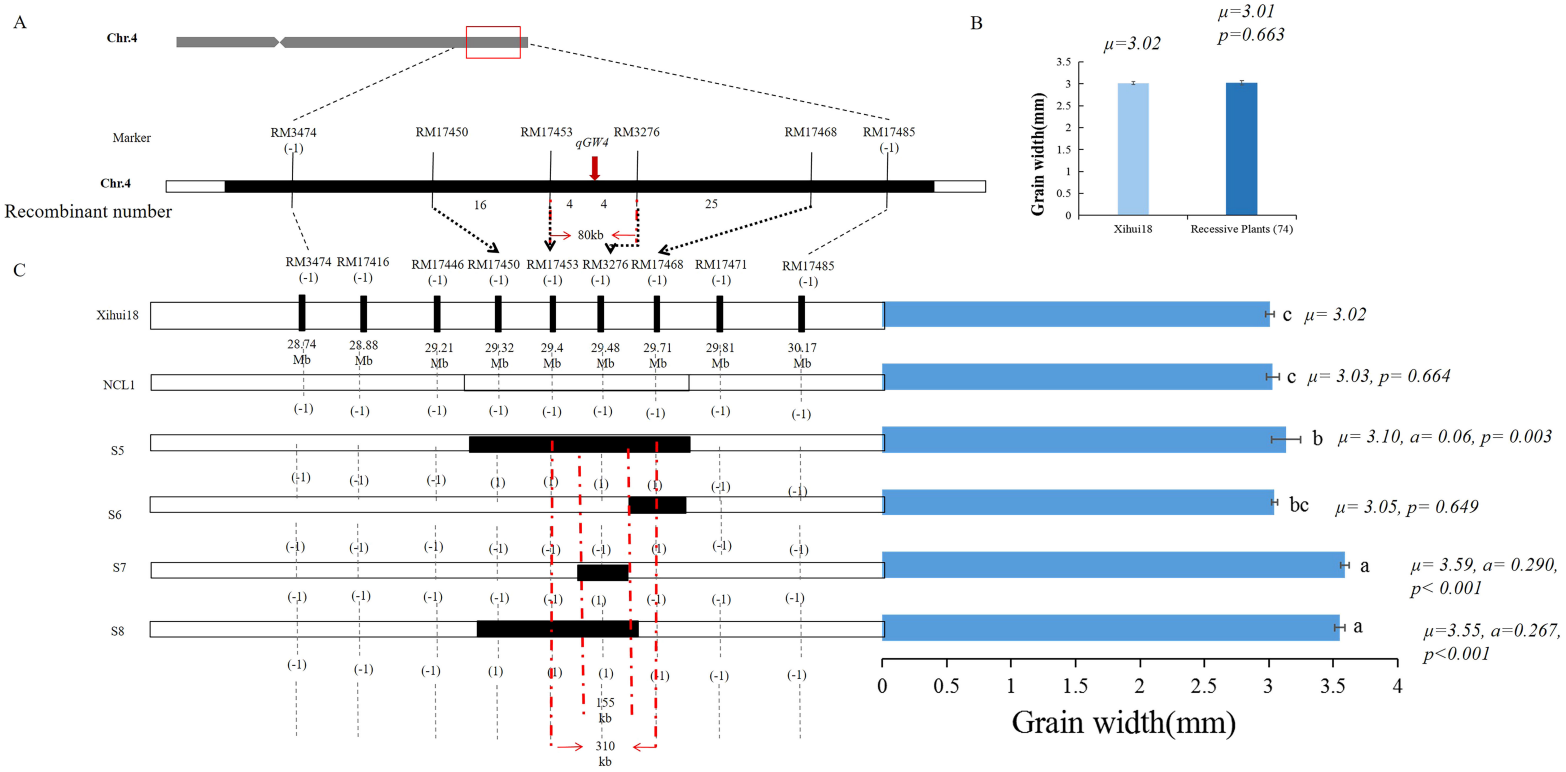
Fine mapping and substitution mapping of *qGW4*. **(A)** Fine-mapping of *qGW4* by linkage analysis. **(B)** Statistic analysis of grain width between Xihui18 (10 plants) and the recessive individuals (74 plants) in the F_3_ population. **(C)** Overlapping substitution mapping of *qGW4*. The black regions represent the estimated substitution length. NCL, negative control line (the lanes of all marker in corresponding substitution intervals same with Xihui18). Different lowercase letters **a**, **b** and **c** on each top column represent existing difference at 0.05 level, which is determined by Duncan’s multiple comparison.

Given the *qGW4* linkage with RM3276 in S1 (**Figure 6A**), *qGW4* was further fine-mapped using 74 recessive plants in the above F_3_ population. The grain width of these recessive plants (3.01 mm) was not significantly different from that of Xihui 18 (3.01 mm) (**Figure 6B**). Within the largest substitution interval of RM17389 and RM17485 of S1, ten SSR markers were designed, seven of which showed polymorphisms between Xihui18 and Jinhui35. Among the seven markers, the bands of three in S1 were the same as those in Jinhui35, indicating that these markers were in the substitution segment, whereas the other four were the same as the recipient Xihui18, suggesting that they belonged to the genetic background of S1. Therefore, by linkage analysis of all markers in the substitution segment using 74 recessive plants, the genetic distances of *qGW4* were 6.20, 2.64, 0.79, and 0.79 cM from RM17468, RM17450, RM7453 and RM3276, respectively. Thus, *qGW4* was fine-mapped between RM17453 and RM3276, with a physical distance of 80 Kb (**Figure 6A**). We also developed a series of secondary SSSLs (S5–S8) for the overlapping substitution mapping of *qGW4* in the F_4_ generation (**Figure 6C**). The grain width (3.03 mm) of the negative control line (NCL), whose lanes of all markers in the substitution fragment were the same as those of Xihui18, displayed no significant difference from that of Xihui18 (3.01 mm). However, S5 carrying the RM17450-RM17453-RM3276-RM17468 substitution segment, S7 containing the RM3276 segment, and S8 harboring the RM17450-RM17453- RM3276 segment exhibited significantly wider grains (3.10, 3.59, and 3.55 mm, respectively) than those of Xihui18 (3.01 mm). Further, S6 carrying the RM17468 segment, showed no difference in grain width (3.05 mm) from that of Xihui18 (3.01 mm). Therefore, *qGW4* should be in 155 kb of the estimated length, and 310 Kb of the largest substitution length of RM17453–RM3276–RM17468 (**Figure 6C**). These results were consistent. *qGW4* should be in 80 Kb of the minimum distance between RM17453 and RM3276, 155 Kb of the estimated length, and 301 kb of the largest substitution length of RM17453–RM3276–RM17468 (**Figures 6A, C**). By predicting the candidate genes within 301 kb of the largest substitution length, four candidate genes of *qGW4* were predicted to encode a MYB protein, DNA-containing protein, PAP fibrinogen family protein, and DUF domain protein (**Table 4**).

**Table 4:** Candidate genes predicted within the *qGW4* fine-mapping interval.

Possible candidate genes	encoding proteins
Candidate gene 1	LOC_Os04g51800 (MYB protein)
Candidate gene 2	LOC_Os04g51794 (DNA binding protein)
Candidate gene 3	LOC_Os04g51792 (PAP fibrinogen family protein)
Candidate gene 4	LOC_Os04g51786 (DUF domain protein)

## 4 Discussion

### 4.1 Eight SSSLs dissected by CSSL-Z708 have promising prospects for enhancing rice yield in breeding application

Rice grain yield is determined by three major “visible” morphological traits: grain weight, grain number per panicle, and panicle number (Li et al., 2021). These yield traits exhibit complex inheritance patterns that are often controlled by several minor QTL. Chromosomal segment substitution lines are ideal materials for the genetic dissection and pyramiding of favorable QTL (Ebitani et al., 2005; Ando et al., 2008; Balakrishnan et al., 2019; Nagata et al., 2023). Here, we characterized a less-grain and wide-grain rice CSSL, Z708, which contained four substitution fragments from Jinhui35 in a Xihui18 background, with an average substitution length of 3.52 Mb.

Although Z708 has some favorable alleles from Jinhui35 for wide and large grains, Its direct use in rice breeding is challenging because of the presence of multiple QTL. To construct more suitable materials for the direct breeding and functional analysis of key genes, eight SSSLs containing various QTL were developed from Z708. As each SSSL has only one difference of a single substitution fragment from its recipient parent, they can identify QTL accurately, which can be verified by the fact that S1-S4 detected more QTL (16) for yield traits than those (7) detected using the F_2_ population of Xihui18/Z708. Many studies have confirmed the high efficiency of QTL detection (Ando et al., 2008; Teng et al., 2012; Ookawa et al., 2016; Zhang et al., 2021a; Hui et al., 2020). Notably, S1, S2, S3, and S4 displayed a significant increase in yield per plant compared to that of Xihui18. For instance, S1 which harbors *qPN4* (a = 0.70), *qGW4* (a = 0.05 mm), *qGWT4* (a = 0.42 g), and *qGPP4* (a = -12.46) exhibited a 17.01% increase in yield per plant relative to Xihui18 (22.30 g); S2 carrying *qGW5* (a = 0.36 mm), *qGWT5* (a = 6.12 g), and *qGPP5* (a = -8.86) showed a 29.4% increase in yield; S3 containing *qPN6* (a = 0.50) and *qGPP6* (a = 9.09) achieved a 33.00% increase, while S4 with *qGPP12* (a = 14.26) increased the yield per plant by 21.40% than that of Xihui18 (22.30 g) (**Figure 4**). Notably, both Xihui18 and Jinhui35 are strong restorer lines carrying restorer genes *Rf1* and *Rf3* on Chr.1, *Rf2* on Chr.2, and *Rf4* on Chr.10 (Akagi et al., 2004; Itabashi et al., 2011; Cai et al., 2013), Thus, some of them could be used as restorer lines to breed new hybrid cultivars by combining with sterile *indica* lines. Therefore, these SSSLs have promising prospects for improving rice yield in breeding by design.

### 4.2 Comparison of QTL for yield traits identified in the study with the reported genes

In total, 16 QTL for yield traits were detected in both the F_2_ population derived from Xihui18/Z708 and the dissected SSSLs. We compared these QTL with previously reported genes located within the corresponding substitution intervals, *qGW4*, *qRLW4*, *qGWT4*, *qPN4*, *qGPP4*, and *qYD4*, all linked to RM3276. Within the substitution interval, *OsAGO2* regulates the distribution of cytokinins by activating *BG3*, thereby increasing rice grain weight, but has little effect on grain width (Yin et al., 2020). Thus, *OsAGO2* may be a candidate gene for *qGWT4*. Determining whether *OsAGO2* is a candidate gene for *qGWT4* requires genetic complementarity studies in the future. Finally, *qGW4* was fine-mapped to 80 Kb of the minimum interval and 301 Kb of the largest substation interval, and four candidate genes were predicted. *MONOCULM 3* encoding *WUSCHEL*, is a key gene involved in the formation of rice tiller buds (Lu et al., 2015). Thus, *MOC3* may be a candidate gene for *qPN4*. *qGW5*, *qRLW5*, *qGWT5*, *qGPP5*, and *qYD5*, all linked to RM2422. *OsGSK2* was found in the substitution interval. *OsGSK2*, a homolog of Arabidopsis *BIN2*, affects cell proliferation and expansion to negatively control grain size by phosphorylating substrates, including *OFP3* (Tong et al., 2012; Xiao et al., 2020). Thus, *OsGSK2* may be a candidate gene for *qGW5*, *qRLW5*, *qGWT5*. Again, *qGW5* and *qGWT5* were also identified as *qGW5* and *qGWT5-2* by Sun et al. using CSSL-Z431 (Sun et al., 2022). However, they came from different donors (Huhan 3), and whether they are different alleles requires further study. *qPN6*, *qGPP6*, and *qYD6* were linked to RM3567. Within the substitution interval of S3, *MOC1/GNP6* regulates tiller and grain number development (Zhang et al., 2021b). Thus, *MOC1/GNP6* is a candidate gene for *qPN6*, *qGPP6*, and *qYD6*. *qGPP12* and *qYD12* were linked to RM1986. Within the substitution interval of S4, *GNP12* encodes *RGH1A* protein and regulates rice yield by regulating panicle length, grain number per panicle, and grain length (Pan et al., 2022). *OsVIL2* improves biomas and grain production by suppressing *OsCKX2* chromatin (Yang et al., 2019). Thus, *GNP12* and OsVIL2 may be candidate genes for *qGPP12* and *qYD12.* However, whether these genes are candidate genes for the related QTL requires further verification using genetic complementarity experiments.

In conclusion, *qGW4*, *qGPP4*, *qYD4*, *qGPP5*, and *qYD5*, which have not been previously reported, provide a good foundation for the functional analysis of these QTLs. Further, S1-S4 carrying favorable alleles, such as *qPN4*, *qGW4*, *qYD4*, *qGW5*, *qGWT5*, *qYD5*, *qPN6*, *qGPP6*, *qYD6*, *qGPP12*, and *qYD12* could be used as new restorer lines for breeding improved hybrid varieties.

## Data Availability

The original contributions presented in the study are included in the article/supplementary material. Further inquiries can be directed to the corresponding author/s.

## References

[B1] Akagi H. Nakamura A. Yokozeki-Misono Y. Inagaki A. Takahashi H. Mori K.. (2004). Positional cloning of the rice *Rf-1* gene, a restorer of BT-type cytoplasmic male sterility that encodes a mitochondria-targeting PPR protein. Theor. Appl. Genet. 108, 1449–1457. doi: 10.1007/s00122-004-1591-2 14968308

[B2] Ali M. L. Sanchez P. L. Yu S.-b. Lorieux M. Eizenga G. C. (2010). Chromosome Segment Substitution Lines: A Powerful Tool for the Introgression of Valuable Genes from *Oryza* Wild Species into Cultivated Rice (*O. sativa*). Rice 3, 218–234. doi: 10.1007/s12284-010-9058-3

[B3] Ando T. Yamamoto T. Shimizu T. Ma X. F. Shomura A. Takeuchi Y.. (2008). Genetic dissection and pyramiding of quantitative traits for panicle architecture by using chromosomal segment substitution lines in rice. Theor. Appl. Genet. 116, 881–890. doi: 10.1007/s00122-008-0722-6 18274726

[B4] Ashikari M. Sakakibara H. Lin S. Y. Yamamoto T. Takashi T. Nishimura A.. (2005). Cytokinin oxidase regulates rice grain production. Science 309, 741–745. doi: 10.1126/science.1113373 15976269

[B5] Balakrishnan D. Surapaneni M. Mesapogu S. Neelamraju S. (2019). Development and use of chromosome segment substitution lines as a genetic resource for crop improvement. Theor. Appl. Genet. 132, 1–25. doi: 10.1007/s00122-018-3219-y 30483819

[B6] Bin Rahman A. N. M. R. Zhang J. (2023). Trends in rice research: 2030 and beyond. Food Energy Secur. 12 (2). doi: 10.1002/fes3.390

[B7] Cai J. Liao Q. P. Dai Z. J. Zhu H. T. Zeng R. Z. Zhang Z. M.. (2013). Allelic differentiations and effects of the *Rf3* and *Rf4* genes on fertility restoration in rice with wild abortive cytoplasmic male sterility. Biol. Plant. 57, 274–280. doi: 10.1007/s10535-012-0294-9

[B8] Chen Q. Tian F. Cheng T. Jiang J. Zhu G. Gao Z.. (2022). Translational repression of FZP mediated by CU-rich element/OsPTB interactions modulates panicle development in rice. Plant J. 110 (5), 1319–1331. doi: 10.1111/tpj.15737 35293072

[B9] Duan P. Rao Y. Zeng D. Yang Y. Xu R. Zhang B.. (2014). *SMALL GRAIN 1*, which encodes a mitogen-activated protein kinase kinase 4, influences grain size in rice. Plant J. 77, 547–557. doi: 10.1111/tpj.12405 24320692

[B10] Ebitani T. Takeuchi Y. Nonoue Y. Yamamoto T. Takeuchi K. Yano M. (2005). Construction and evaluation of chromosome segment substitution lines carrying overlapping chromosome segments of *indica* rice cultivar ‘Kasalath’ in a genetic background of *japonica* elite cultivar ‘Koshihikari’. Breed. Sci. 55, 65–73. doi: 10.1270/jsbbs.55.65

[B11] Fan C. H. Xing Y. Z. Mao H. L. Lu T. T. Han B. Xu C. G.. (2006). *GS3*, a major QTL for grain length and weight and minor QTL for grain width and thickness in rice, encodes a putative transmembrane protein. Theor. Appl. Genet. 112, 1164–1171. doi: 10.1007/s00122-006-0218-1 16453132

[B12] Hao J. Q. Wang D. K. Wu Y. B. Huang K. Duan P. G. Li N.. (2021). The GW2-WG1-OsbZIP47 pathway controls grain size and weight in rice. Mol. Plant. 14(8), 1266–1280. doi: 10.1016/j.molp.2021.04.011 33930509

[B13] Huang X. Qian Q. Liu Z. Sun H. He S. Luo D. Xia G.. (2009). Natural variation at the DEP1 locus enhances grain yield in rice. Nat. Genet. 41 (4), 494–497. doi: 10.1038/ng.352 19305410

[B14] Huang Y. Y. Zhao S. S. Fu Y. C. Sun H. D. Ma X. Tan L. B.. (2018). Variation in the regulatory region of FZP causes increases in secondary inflorescence branching and grain yield in rice domestication. Plant J. 96 (4), 716–733. doi: 10.1111/tpj.14062 30101570

[B15] Hui W. Jiayu Z. Farkhanda N. Juan L. Shuangfei S. Guanghua H.. (2020). Identification of rice QTLs for important agronomic traits with long-kernel CSSL-Z741 and three SSSLs. Rice Sci. 27, 414–422. doi: 10.1016/j.rsci.2020.04.008

[B16] Ishimaru K. Hirotsu N. Madoka Y. Murakami N. Hara N. Onodera H.. (2013). Loss of function of the IAA-glucose hydrolase gene *TGW6* enhances rice grain weight and increases yield. Nat. Genet. 45, 707–70+. doi: 10.1038/ng.2612 23583977

[B17] Itabashi E. Iwata N. Fujii S. Kazama T. Toriyama K. (2011). The fertility restorer gene, *Rf2*, for Lead Rice-type cytoplasmic male sterility of rice encodes a mitochondrial glycine-rich protein. Plant J. 65, 359–367. doi: 10.1111/j.1365-313X.2010.04427.x 21265890

[B18] Jiang P. F. Wang S. L. Jiang H. Y. Cheng B. J. Wu K. Q. Ding Y. (2018). The COMPASS-Like Complex Promotes Flowering and Panicle Branching in Rice. Plant Physiology 176 (4), 2761–2771. doi: 10.1104/pp.17.01749 29440594 PMC5884598

[B19] Kumari J. Lakhwani D. Jakhar P. Sharma S. Tiwari S. Mittal S.. (2023). Association mapping reveals novel genes and genomic regions controlling grain size architecture in mini core accessions of Indian National Genebank wheat germplasm collection. Front. Plant Sci. 14. doi: 10.3389/fpls.2023.1148658 PMC10345843 37457353

[B20] Li G. Tang J. Zheng J. Chu C. (2021). Exploration of rice yield potential: Decoding agronomic and physiological traits. Crop J. 9 (3), 577–589. doi: 10.1016/j.cj.2021.03.014

[B21] Li X. Y. Qian Q. Fu Z. M. Wang Y. H. Xiong G. S. Zeng D. L.. (2003). Control of tillering in rice. Nature 422 (6932), 618–621. doi: 10.1038/nature01518 12687001

[B22] Li Z. Riaz A. Zhang Y. Anis G. B. Zhu A. Cao L.. (2019). Quantitative trait loci for rice yield-related traits using chromosomal segment substitution lines. Rice Sci. 26 (5), 261–264. doi: 10.1016/j.rsci.2019.02.001

[B23] Liu J. F. Chen J. Zheng X. M. Wu F. Q. Lin Q. B. Heng Y. Q.. (2017). *GW5* acts in the brassinosteroid signalling pathway to regulate grain width and weight in rice. Nat. Plants 3 (5). doi: 10.1038/nplants.2017.43 28394310

[B24] Liu Q. Han R. Wu K. Zhang J. Ye Y. Wang S.. (2018). G-protein βγ subunits determine grain size through interaction with MADS-domain transcription factors in rice. Nat. Commun. 9. doi: 10.1038/s41467-018-03047-9 PMC5829230 29487282

[B25] Li G. L. Xu B. X. Zhang Y. P. Xu Y. W. Khan N. U. Xie J. Y.. (2022). *RGN1* controls grain number and shapes panicle architecture in rice. Plant Biotechnol. J. 20 (1), 158–167. doi: 10.1111/pbi.13702 34498389 PMC8710824

[B26] Lu Z. Shao G. Xiong J. Jiao Y. Wang J. Liu G.. (2015). *MONOCULM 3*, an ortholog of *WUSCHEL* in rice, is required for tiller bud formation. J. Genet. Genomics 42 (2), 71–78. doi: 10.1016/j.jgg.2014.12.005 25697101

[B27] Ma M. Shen S. Y. Bai C. Wang W. Q. Feng X. H. Ying J. Z.. (2023). Control of grain size in rice by TGW3 phosphorylation of OsIAA10 through potentiation of OsIAA10-OsARF4-mediated auxin signaling. Cell Rep. 42 (3), 112187. doi: 10.1016/j.celrep.2023.112187 36871218

[B28] Maurer M. J. Sutardja L. Pinel D. Bauer S. Muehlbauer A. L. Ames T. D.. (2017). Quantitative trait loci (QTL)-guided metabolic engineering of a complex trait. ACS Synth. Biol. 6, 566–581. doi: 10.1021/acssynbio.6b00264 27936603

[B29] McCouch S. R. Kochert G. Yu Z. H. Wang Z. Y. Khush G. S. Coffman W. R.. (1988). Molecular mapping of rice chromosomes. Theor. Appl. Genet. 76, 815–829. doi: 10.1007/bf00273666 24232389

[B30] McKenzie K. S. Rutger J. N. (1983). Genetic-analysis of amylose content, alkali spreading score, and grain dimensions in rice. Crop Sci. 23, 306–313. doi: 10.2135/cropsci1983.0011183X002300020031x

[B31] Nagata K. Nonoue Y. Matsubara K. Mizobuchi R. Ono N. Shibaya T.. (2023). Development of 12 sets of chromosome segment substitution lines that enhance allele mining in Asian cultivated rice. Breed. Sci. 73, 332–342. doi: 10.1270/jsbbs.23006 37840983 PMC10570878

[B32] Ookawa T. Aoba R. Yamamoto T. Ueda T. Takai T. Fukuoka S.. (2016). Precise estimation of genomic regions controlling lodging resistance using a set of reciprocal chromosome segment substitution lines in rice. Sci. Rep. 6 (1). doi: 10.1038/srep30572 PMC4964586 27465821

[B33] Pan Y.-H. Chen L. Guo H.-F. Feng R. Lou Q.-J. Rashid M. A. R.. (2022). Systematic analysis of NB-ARC gene family in rice and functional characterization of *GNP12* . Front. Genet. 13. doi: 10.3389/fgene.2022.887217 PMC9244165 35783267

[B34] Paterson A. H. Damon S. Hewitt J. D. Zamir D. Rabinowitch H. D. Lincoln S. E.. (1991). Mendelian factors underlying quantitative traits in tomato - comparison across species, generations, and environments. Genetics 127, 181–197. doi: 10.1093/genetics/127.1.181 1673106 PMC1204303

[B35] Rezvi H. U. A. Tahjib-Ul-Arif M. Azim M. A. Tumpa T. A. Tipu M. M. H. Najnine F.. (2023). Rice and food security: Climate change implications and the future prospects for nutritional security. Food Energy Secur. 12 (1). doi: 10.1002/fes3.430

[B36] Ruan B. Shang L. Zhang B. Hu J. Wang Y. Lin H.. (2020). Natural variation in the promoter of TGW2 determines grain width and weight in rice. Plant J. 227 (2), 430. doi: 10.1111/nph.16540 32167575

[B37] Shabir G. Aslam K. Khan A. R. Shahid M. Manzoor H. Noreen S.. (2017). Rice molecular markers and genetic mapping: Current status and prospects. J. Integr. Agric. 16, 1879–1891. doi: 10.1016/s2095-3119(16)61591-5

[B38] Shalmani A. Ullah U. Tai L. Zhang R. Jing X.-Q. Muhammad I.. (2023). OsBBX19-OsBTB97/OsBBX11 module regulates spikelet development and yield production in rice. Plant Sci. 334, 111779. doi: 10.1016/j.plantsci.2023.111779 37355232

[B39] Shomura A. Izawa T. Ebana K. Ebitani T. Kanegae H. Konishi S.. (2008). Deletion in a gene associated with grain size increased yields during rice domestication. Nat. Genet. 40, 1023–1028. doi: 10.1038/ng.169 18604208

[B40] Si L. Chen J. Huang X. Gong H. Luo J. Hou Q.. (2016). *OsSPL13* controls grain size in cultivated rice. Nat. Genet. 48, 447–44+. doi: 10.1038/ng.3518 26950093

[B41] Song X.-J. Huang W. Shi M. Zhu M.-Z. Lin H.-X. (2007). A QTL for rice grain width and weight encodes a previously unknown RING-type E3 ubiquitin ligase. Nat. Genet. 39, 623–630. doi: 10.1038/ng2014 17417637

[B42] Sun S. Wang Z. Xiang S. Lv M. Zhou K. Li J.. (2022). Identification, pyramid, and candidate gene of QTL for yield-related traits based on rice CSSLs in *indica* Xihui18 background. Mol. Breed. 42 (4). doi: 10.1007/s11032-022-01284-x PMC10248596 37309460

[B43] Sun L. Q. Bai Y. Wu J. Fan S. J. Chen S. Y. Zhang Z. Y.. (2024). OsNLP3 enhances grain weight and reduces grain chalkiness in rice. Plant Commun. 5 (10). doi: 10.1016/j.xplc.2024.100999 PMC11574284 38853433

[B44] Surapaneni M. Balakrishnan D. Mesapogu S. Addanki K. R. Yadavalli V. R. Venkata T. V. G. N.. (2017). Identification of major effect QTLs for agronomic traits and CSSLs in rice from swarna/*oryza nivara* derived backcross inbred lines. Front. Plant Sci. 8. doi: 10.3389/fpls.2017.01027 PMC5480306 28690618

[B45] Teng B. Zeng R. Wang Y. Liu Z. Zhang Z. Zhu H.. (2012). Detection of allelic variation at the *Wx* locus with single-segment substitution lines in rice (*Oryza sativa* L.). Mol. Breed. 30, 583–595. doi: 10.1007/s11032-011-9647-x

[B46] Tong H. Liu L. Jin Y. Du L. Yin Y. Qian Q.. (2012). DWARF AND LOW-TILLERING acts as a direct downstream target of a GSK3/SHAGGY-like kinase to mediate brassinosteroid responses in rice. Plant Cell 24, 2562–2577. doi: 10.1105/tpc.112.097394 22685166 PMC3406904

[B47] Weng J. Gu S. Wan X. Gao H. Guo T. Su N.. (2008). Isolation and initial characterization of *GW5*, a major QTL associated with rice grain width and weight. Cell Res. 18, 1199–1209. doi: 10.1038/cr.2008.307 19015668

[B48] Wu Y. Wang Y. Mi X.-F. Shan J.-X. Li X.-M. Xu J.-L.. (2016). The QTL GNP1 encodes GA20ox1, which increases grain number and yield by increasing cytokinin activity in rice panicle meristems. PloS Genet. 12 (10). doi: 10.1371/journal.pgen.1006386 PMC5072697 27764111

[B49] Xiao Y. Zhang G. Liu D. Niu M. Tong H. Chu C. (2020). GSK2 stabilizes OFP3 to suppress brassinosteroid responses in rice. Plant J. 102, 1187–1201. doi: 10.1111/tpj.14692 31950543

[B50] Xu G. Deng K. Yu J. Li Q. Li L. Xiang A.. (2023). Genetic effects analysis of QTLs for rice grain size based on CSSL-Z403 and its dissected single and dual-segment substitution lines. Int. J. Mol. Sci. 24 (15). doi: 10.3390/ijms241512013 PMC10418668 37569388

[B51] Yang J. Cho L.-H. Yoon J. Yoon H. Wai A. H. Hong W.-J.. (2019). Chromatin interacting factor OsVIL2 increases biomass and rice grain yield. Plant Biotechnol. J. 17, 178–187. doi: 10.1111/pbi.12956 29851259 PMC6330541

[B52] Yang W. Liang J. Hao Q. Luan X. Tan Q. Lin S.. (2021). Fine mapping of two grain chalkiness QTLs sensitive to high temperature in rice. Rice 14, 1–10. doi: 10.1186/s12284-021-00476-x 33792792 PMC8017073

[B53] Yin W. Xiao Y. Niu M. Meng W. Li L. Zhang X.. (2020). ARGONAUTE2 enhances grain length and salt tolerance by activating *BIG GRAIN3* to modulate cytokinin distribution in rice. Plant Cell 32, 2292–2306. doi: 10.1105/tpc.19.00542 32409321 PMC7346564

[B54] Zhang G. (2021a). Target chromosome-segment substitution: A way to breeding by design in rice. Crop J. 9, 658–668. doi: 10.1016/j.cj.2021.03.001

[B55] Zhang Q. Wu R. Hong T. Wang D. Li Q. Wu J.. (2024). Natural variation in the promoter of qRBG1/OsBZR5 underlies enhanced rice yield. Nat. Commun. 15, 8565–8565. doi: 10.1038/s41467-024-52928-9 39362889 PMC11449933

[B56] Zhang J. Lin Q. Wang X. Shao J. Ren Y. Liu X.. (2025). The DENSE AND ERECT PANICLE1-GRAIN NUMBER ASSOCIATED module enhances rice yield by repressing CYTOKININ OXIDASE 2 expression. Plant Cell 37 (1), koae309. 10.1093/plcell/koae309 PMC11663551 39660553

[B57] Zhang Z. Sun X. Ma X. Xu B. Zhao Y. Ma Z.. (2021b). *GNP6*, a novel allele of *MOC1*, regulates panicle and tiller development in rice. Crop J. 9 (1), 57–67. doi: 10.1016/j.cj.2020.04.011

[B58] Zhao F. M. Tan Y. Zheng L. Y. Zhou K. He G. H. Ling Y. H.. (2016). Identification of rice chromosome segment substitution line Z322-1-10 and mapping QTLs for agronomic traits from the F_3_ population. Cereal Res. Commun. 44, 370–380. doi: 10.1556/0806.44.2016.022

[B59] Zhao L. Tan L. Zhu Z. Xiao L. Xie D. Sun C. (2015). *PAY1* improves plant architecture and enhances grain yield in rice. Plant J. 83, 528–536. doi: 10.1111/tpj.12905 26095647 PMC4758413

[B60] Zhong H. Liu C. Kong W. Zhang Y. Zhao G. Sun T.. (2020). Effect of multi-allele combination on rice grain size based on prediction of regression equation model. Mol. Genet. Genomics 295, 465–474. doi: 10.1007/s00438-019-01627-y 31863176

[B61] Zhu W. Lin J. Yang D. Zhao L. Zhang Y. Zhu Z.. (2009). Development of chromosome segment substitution lines derived from backcross between two sequenced rice cultivars, *indica* recipient 93-11 and *japonica* donor nipponbare. Plant Mol. Biol. Rep. 27, 126–131. doi: 10.1007/s11105-008-0054-3

